# Coronavirus Disease 19 from the Perspective of Ageing with Focus on Nutritional Status and Nutrition Management—A Narrative Review

**DOI:** 10.3390/nu13041294

**Published:** 2021-04-14

**Authors:** Elisabet Rothenberg

**Affiliations:** Faculty of Health Science, Kristianstad University, 291 88 Kristianstad, Sweden; elisabet.rothenberg@hkr.se; Tel.: +46-706-41-45-81

**Keywords:** COVID-19, SARS-CoV-2, older adults, inflammation, malnutrition, nutrition, vitamin D, sarcopenia, olfactory and gustatory dysfunction, GLIM

## Abstract

The novel severe acute respiratory syndrome coronavirus (COVID-19) has hit older adults harder due to a combination of age-related immunological and metabolic alterations. The aim of this review was to analyze the COVID-19 literature with respect to nutritional status and nutrition management in older adults. No studies only on people aged 65+ years were found, and documentation on those 80+ was rare. Age was found to be strongly associated with worse outcomes, and with poor nutritional status. Prevalence of malnutrition was high among severely and critically ill patients. The studies found a need for nutrition screening and management, and for nutrition support as part of follow-up after a hospital stay. Most tested screening tools showed high sensitivity in identifying nutritional risk, but none were recognized as best for screening older adults with COVID-19. For diagnosing malnutrition, the Global Leadership Initiative on Malnutrition (GLIM) criteria are recommended but were not used in the studies found. Documentation of olfactory and gustatory dysfunction in relation to nutritional status is missing in older adults. Other COVID-19-associated factors with a possible impact on nutritional status are poor appetite and gastrointestinal symptoms. Vitamin D is the nutrient that has attracted the most interest. However, evidence for supplementation of COVID-19 patients is still limited and inconclusive.

## 1. Introduction

The severe acute respiratory syndrome coronavirus 2 (SARS-CoV-2) was first identified in Wuhan, Hubei province, China, in December 2019 as the etiologic agent of a new type of viral pneumonia, the Coronavirus Disease 19 (COVID-19). On 11 March 2020, it was declared as a pandemic by the World Health Organization (WHO) [1] and created a situation that placed completely new demands on behavior. Social distancing has become a core value in public places. Social interactions in all forms, from between single individuals to bilateral relations between countries, have changed. Age is by far the strongest predictor of a fatal outcome, with those aged over 70 years showing a significant increase in mortality [2].

The aim of the present review was to analyze the current COVID-19 literature from the perspective of ageing with a focus on nutritional status and nutrition management including vitamin D and the effect of olfactory and gustatory dysfunction on nutritional status.

## 2. Materials and Methods

This is a narrative review.

### 2.1. Literature Search Strategy

Electronic bibliographic databases PubMed SARS-CoV-2 Data, Scopus, and Web of Science were used for conducting this literature search. The search strategy was based on a clear and careful selection of key words and terms. After repeated attempts and adjustments, the final search strategies were built as follows:

(a) COVID-19 AND (Malnutrition OR Undernutrition OR Nutrition risk OR Body Composition OR Muscle mass OR Lean body mass);
(b) COVID-19 AND (vitamin D OR vitamin D deficiency);
(c) COVID-19 AND gustatory AND olfactory AND nutritional.

The search included all studies from the beginning of publication on COVID-19 until 13 January 2021. The search filter was set on human beings, language was restricted to English, and full text paper and search terms should be present in the abstract. The types of studies included were: clinical study, clinical trial, multicenter study, observational study, randomized control study, meta-analysis, and systematic review. References from reviews and systematic reviews (SRs) were also checked manually for further screening in case they were not identified during the whole search process. References in the reference list marked with * are the result of the database search.

### 2.2. Inclusion and Exclusion Criteria

Older people are defined as people aged 65 years and over according to the Eurostat definition of old age [3]; however, no studies were found that only included people over this age. Therefore, studies targeting people of middle age or older were included and/or studies in which data on older adults were specifically presented. The focus was on: (a) nutritional status and nutrition management, for instance, studies including measurement of nutritional status defined by screening tools or by measurement of body composition; (b) research that included olfactory and gustatory dysfunction and nutritional risk; and (c) vitamin D intake and status. Studies were excluded if the target group did not include older adults.

## 3. The Role of Inflammation

A main cause of death in COVID-19 is abnormal immune response with uncontrolled secretion of cytokines, the so-called a cytokine storm [4]. Immunosenescence is a complex process characterized by poor response to vaccines, a higher incidence of infections, and the prevalence of chronic conditions such as hypertension, cardiovascular disease, and chronic obstructive pulmonary disease related to ageing [5,6,7]. The balance between systemic proinflammatory and anti-inflammatory cytokines changes toward a dominance of the proinflammatory cytokines with risk of permanent mild inflammation called inflamm-aging [8,9,10]. Several pathophysiologic alterations are involved, such as changes in angiotensin-converting enzyme 2 (ACE2) receptor expression [8], increases in oxidative stress, an accumulation in adipose tissue and immune senescent cells, and vitamin D deficiency [8]. The underlying mechanisms are still unclear.

Obesity, which is prevalent in higher ages [11,12], increases the infiltration of fat into muscle, leading to insulin resistance and enhanced secretion of proinflammatory myokines, which may exacerbate adipose tissue inflammation, thereby supporting chronic low-grade systemic inflammation, disturbing protective immunity, which affects susceptibility to infections and lowers the antibody response to vaccinations, including influenza [13]. Furthermore, obesity complicates muscle protein synthesis and lowers physical function [14,15].

## 4. Muscle Protein Metabolism

Ageing is associated with impaired muscle protein metabolism mainly related to: inadequate intake, a reduced ability to use available protein, and a greater need for protein [16]. Proinflammatory cytokines (TNF, IL1, and IL6), as part of inflamm-aging, cause increasing degradation [17] and decreasing synthesis [18] of body proteins [19,20], mainly muscle protein. This leads to lower muscle strength [20] and limited amino acid storage [21] with poorer defense against metabolic stresses such as COVID-19 infection [22].

## 5. Sarcopenia

Sarcopenia is mainly associated with ageing due to its associated impaired muscle protein synthesis [23,24], but sarcopenia is also dependent on the levels of peak muscle mass and strength attained earlier in life [25]. Among underlying factors are a sedentary lifestyle, poor diet, bed rest, and immobility [23]. Sarcopenia might be a consequence of COVID-19, but also a risk factor for a worse outcome related to limited amino acid storage [21]. In this context, sarcopenic obesity [12], meaning low muscle mass in combination with excessive fat mass, could be of special interest as obesity is regarded as an independent risk factor for worse outcomes of COVID-19 [26].

## 6. Body Composition

To date, only a few studies have reported on body composition (BC) in COVID-19 patients [27,28,29]. In an observational and cross-sectional cohort study from the Netherlands, 54 patients, 30 in the ward and 24 in the Intensive Care Unit (ICU), were investigated. The mean age was 67 years, 37% women, and body mass index (BMI) of 29.7 kg/m^2^ (95% CI 28.2–31.1). BC was evaluated with bioelectric impedance (BIA). No differences were found related to BC, including fat mass, visceral fat area, and fat-free mass, nor were differences in disease severity found between those at the ward and those in the ICU [27]. However, according to the BMI data, the population was quite homogeneous. Furthermore, BC based on BIA must be interpreted with caution in critically-ill patients. It is sensitive to fluid overload and disturbances in the distribution of extra- and intracellular water [30]. Interestingly, a lower phase angle was associated with increased odds of severe COVID-19 [27]. Phase angle uses raw impedance data and provides information on hydration status, body cell mass, and cell integrity, without algorithm-inherent errors or assumptions such as constant tissue hydration. It was proven to have a prognostic impact regarding impaired nutritional and functional status and mortality [31].

In a retrospective study from Wuhan, China, 143 hospitalized patients were investigated [28], of whom 45 were identified as critically ill. The median age was 66 with interquartile range (IQR) (56–73.5) years, 51% were women. BC was measured with abdominal computed tomography (CT). No significant difference in age, sex, or BMI (median 23.4 kg/m^2^ with IQR (21.9–25.3)) was found between the critically-ill patients and the rest. The authors concluded that COVID-19 patients with visceral adiposity or high intramuscular fat deposition have a higher risk of critical illness [28].

In a multicenter pilot, the aim was to investigate whether the ratio of fat-to-muscle area, measured by low-dose computed tomography, can predict severe progression of COVID-19 within a follow-up period [29]. In total, 58 individuals, 21 women, mean age 59.3 (SD ± 16.2) years, were included. It was found that in addition to age, low muscle in conjunction with a high fat area represented prognostic information related to the outcome and the need for ICU treatment.

## 7. Malnutrition

Malnutrition is characterized by weight loss, muscle wasting, increased risk of infections, long length of hospital stay, mortality, and decreased quality of life, and has significant costs for society [32,33,34,35]. ICU stay, multimorbidity, and older age are factors associated with a high risk and incidence of malnutrition [33,36,37]. Severe hyperinflammation, mechanical ventilation, and a prolonged hospital stay could further increase the risk [38,39]. Importantly, patients who are overweight or obese can be classified as malnourished according to the Global Leadership Initiative on Malnutrition (GLIM) criteria, [40].

## 8. COVID-19 in Old Age

**Figure 1:**
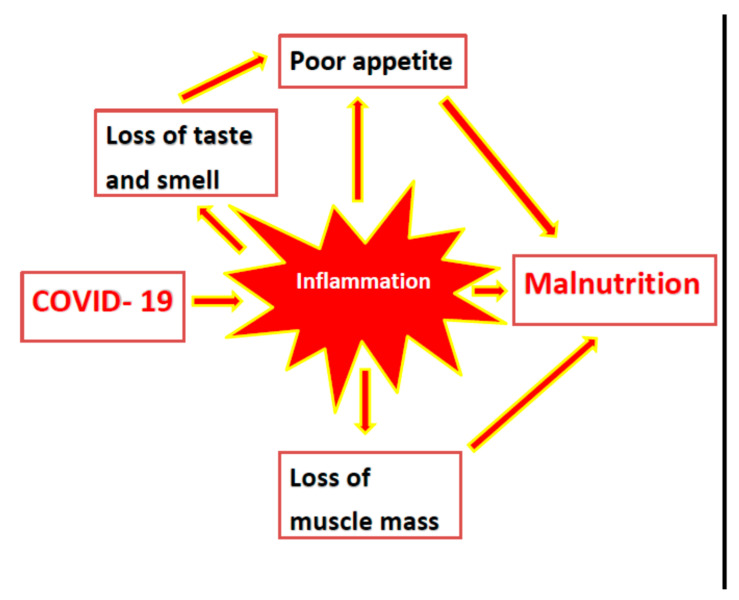
Risk factors for malnutrition that are related to the novel severe acute respiratory syndrome coronavirus (COVID-19).

**Figure 2:**
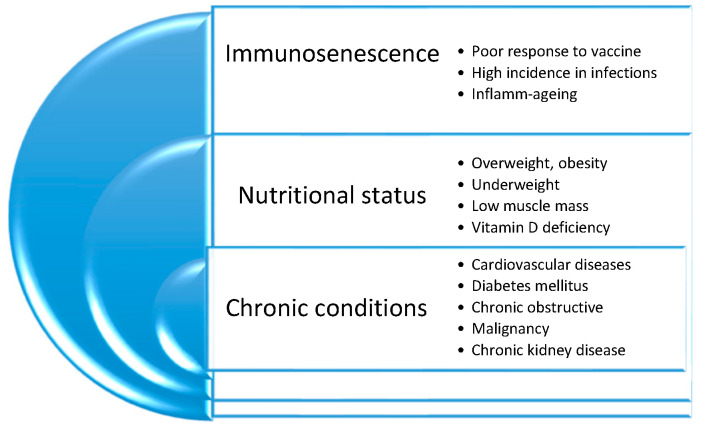
COVID-19 in old age risk factors.

The combination of inflamm-aging, muscle protein degradation, high prevalence of sarcopenia with or without obesity, multi-morbidity, and malnutrition makes older adults additionally vulnerable to COVID-19, as shown in Figure 1. This is both in terms of the risk of being infected and the course of the disease and its outcome [26], as shown in Figure 2. The literature is limited; nevertheless, there are some studies that evaluated nutritional status in older COVID-19 patients [41,42,43,44,45,46,47].

In a cross-sectional study by Li et al. from Wuhan, China, nutritional status in older inpatients was evaluated using the Mini Nutritional Assessment (MNA) [41]. In total, 182 patients were included, 64% women, mean age 68.5 ± 8.8 years. In total, 19% were classified as being without malnutrition (BMI 25.6 ± 3.0 kg/m^2^), 28% classified as at risk (BMI 23.3 ± 3.4 kg/m^2^), and 53% classified as malnourished (BMI 21.1 ± 3.6 kg/m^2^). The presence of comorbid diabetes varied between groups with the highest prevalence in the malnourished group. BMI, calf circumference, albumin, and hemoglobin also differed between groups, with the lowest values in the malnourished group. Diabetes, low calf circumference, and low albumin were independent risk factors for malnutrition. The authors argued that a reason why the overall prognosis was worse in older adults may be the poor nutritional status that is related to muscle protein break down for synthesis of factors involved in the inflammatory response of COVID-19 as C-reactive protein (CRP), ferritin, tumor necrosis factor alpha, interleukin family factors, etc. [48]. Angiotensin-converting enzyme 2 is highly expressed in the gastrointestinal tract [49], which is therefore a target for infection [50], and it may be a mediator of symptoms such as diarrhea, nausea, and vomiting that exacerbate malnutrition [51]. As part of the inflammatory response appetite is also affected [52]. The authors concluded that prevalence of malnutrition was high, and nutritional support should be strengthened during treatment, especially for those with diabetes, low calf circumference, or low albumin [41].

The study Zhao et al. in Wuhan, China, was a retrospective, observational study of patients identified as severely or critically ill with COVID-19. Nutrition risk was assessed using the Nutritional Risk Screening 2002 method (NRS) [42]. In total, 413 patients were included, 49% women, mean age 60.3 ± 12.7 years. Of 346, 67 were found to be severely and critically ill. A higher proportion of critically ill were found with high NRS scores, which were related to inflammation- and nutrition-related markers. These patients also were the oldest, showed the highest mortality rate at 47%, and had the highest risk of having a longer stay in hospital. In total, 35% were overweight, and 9% obese, but no difference was found between BMI groups (mean 23.7) in relation to the severity of illness. Interestingly, among those with NRS score ≥3, only 84 (of 342, 25%) received nutrition support. The authors concluded that those with higher NRS scores have worse outcomes and require nutrition therapy.

The study of Zhang et al. in Wuhan, China, was also retrospective and observational, and included 136 critically-ill patients in the ICU, 37% women, median age 69 years (IQR: 57–77) [43]. BMI was not reported. Nutrition risk was assessed using the Nutrition Risk in the Critically Ill (mNUTRIC) score, and 61% scored as high risk. These patients were also older and showed a higher mortality rate compared with those at low risk. In total, 57% received enteral nutrition, 10% total parenteral nutrition, 22% a combination, 11% did not receive any nutritional support as a result of contraindications, and 47% received oral feeding. Enteral nutrition intolerance was expressed as vomiting or gastric retention in 32%, and hyperglycemia in 63%. The authors concluded that patients with high nutritional risk at ICU admission exhibited significantly higher mortality than those with low nutritional risk.

The study of Li et al. was a multicenter retrospective observational study in Wuhan, China [44]. In total, 523 patients were recruited, with 377 classified as severe and 146 classified as critically ill, 52% women, and mean age 54.2 ± 15.9 years. In total, 40% were admitted to ICU and 22% died. Patients admitted to ICU had lower BMI (21.7) and plasma proteins. Those ICU patients who died were the oldest (69.2 ± 11.4 years), had the lowest plasma proteins, and the highest nutritional risk score. The proportion receiving parenteral nutrition was higher in nonsurvivors than in survivors, and the time to start nutrition therapy was longer. After adjusting for age and sex, each SD increase in BMI reduced the risk of in-hospital death by 13%, and the risk of ICU transfer by 7%. The in-hospital survival time of patients with albumin level ≤35 g/L was significantly decreased. The authors concluded that severe and critical patients have a high risk of malnutrition. Early nutritional risk screening and therapy for COVID-19 patients are necessary.

The study by Di Filippo et al. was a post hoc analysis of a prospective observational cohort study of patients discharged home from either a medical ward or an emergency department in Italy. Patients were re-evaluated after remission at the Outpatient COVID-19 Follow-Up Clinic after approximately 3 weeks [45]. A total of 213 patients were included: 33% women, median age 59.0 years (IQR 49.5; 67.9), and median BMI of 24.7; 31.0). In total, 70% were overweight or obese at baseline, and only 2% were underweight. BMI did not differ between the 73% who became hospitalized compared with those non-hospitalized. Hospitalized patients had the highest mean age. In total, 29% of all patients, and 31% of hospitalized patients vs. 21% of those managed at home, lost >5% of their initial body weight (median loss 8.1%). Those who had lost weight had greater systemic inflammation, impaired renal function, and longer disease duration compared with those whose weight was stable. Only disease duration independently predicted weight loss. The authors concluded that COVID-19 might negatively impact body weight and nutritional status, indicating a need for nutritional evaluation, counselling, and treatment throughout the course of the disease, and after clinical remission.

In an observational longitudinal study by Bedock et al., the aim was to investigate the links between malnutrition and disease severity, and the impact of malnutrition on outcomes such as the need for ICU care or death [46]. Malnutrition was diagnosed in accordance with the GLIM criteria [40]. A total of 160 patients were admitted from the emergency department, medical units, or the ICU. Complete nutritional data were available for 114 subjects, average age 59.9 ± 15.9 years (40% women). Those without complete nutritional data were older. The overall prevalence of malnutrition was 42% (moderate disease severity: 24%, severe disease severity: 18%) and 67% in those admitted from the ICU. No association was found between nutritional status and comorbidity, clinical signs of COVID-19, risk of transfer to ICU, or death. The main nutritional parameters that differed were BMI and % of weight loss (*p* < 0.01), with those severely malnourished having the lowest BMI and the highest proportion of weight loss compared with those not malnourished or those who were moderately malnourished. The authors concluded that the results underline the value of nutritional risk screening and the need for early nutritional management in COVID-19 patients.

The study by De Lorenzo et al. was a retrospective and prospective cohort study [47]. The aims were to investigate whether COVID-19 leaves behind residual dysfunction, and to identify patients who might benefit from post-discharge follow-up, defined as, among other factors, those patients with malnutrition. A total of 185 patients were included, with a median age of 57 years (IQR 48; 67). In total, 59% needed follow-up, and 63% were at risk of malnutrition or found to be malnourished. As well as BMI, age emerged as an independent predictor of a need for follow-up. Patients who were >63 years old, or those who were <63 years and had noninvasive ventilation or diabetes, had the highest probability of needing follow-up. The authors concluded that COVID-19 may produce residual physical and psychological dysfunctions. Multidisciplinary follow-up was therefore crucial to avoid a second wave of late health problems associated with this pandemic.

In summary, these studies showed, in line with earlier findings, that age is strongly associated with worse outcomes. However, none of them were conducted only on people over the age 65 years, and information on the oldest population group (80+ years) was missing. Prevalence of malnutrition was high among severely- and critically-ill patients. Nevertheless, the relationship between malnutrition and COVID-19 might be bidirectional, with the infection potentially causing severe malnutrition at the same time as malnutrition negatively affecting prognosis. All seven studies call for the need for nutrition management and support. However, none used the GLIM criteria described below to diagnose malnutrition [40].

## 9. Olfactory and Gustatory Dysfunction

The association between olfactory disturbances and common viral upper respiratory tract infections is well-established, and similar disturbances of taste have also been reported with COVID-19 [53,54]. Normally symptoms regress, but they could persist for a longer time, presumably due to direct olfactory insult by the virus. With regard to individual studies, the prevalence range is widely related to differences in age, severity of disease, setting, different assessment methods, and study design [55,56]. Higher prevalence has been reported in studies with objective assessment methods compared with subjective methods, as patients might be unaware of their dysfunction leading to a possible underestimation of the problem [57,58].

Two systematic reviews specifically addressed the effect of age and found lower prevalence with increasing age [55,57]. A potential explanation is that olfactory and gustatory dysfunction may be related to a milder course of the infection [59] and older adults are more likely to experience severe COVID-19 infection and more severe symptoms such as respiratory distress. Old age per se is associated with impairments in the olfactory and gustatory system [60]. If COVID-19 aggravates the situation, it might be a risk factor for malnutrition. However, this is still unknown, as no studies were found that examined olfactory and gustatory dysfunction in relation to malnutrition.

## 10. Nutrition Management

### 10.1. Nutritional Screening of COVID-19 Patients

A recent SR studied screening tools aimed at assessing nutritional risk in older COVID-19 patients and to clarify their measurement properties [61]. Four studies, all conducted in China, were analyzed. Sample sizes ranged from 6–182 individuals aged 65–87 years. Seven nutritional screening and assessment tools were used: the Nutritional Risk Screening 2002 (NRS-2002), the Mini Nutritional Assessment (MNA), the MNA short form (MNA-SF), the Malnutrition Universal Screening Tool (MUST), the Nutritional Risk Index (NRI), the Geriatric NRI (GNRI), and a modified Nutrition Risk in the Critically ill (mNUTRIC) score. Nutritional risk varied between 27% and 100% of individuals. Most tools showed high sensitivity in identifying nutritional risk, but none were recognized as the best for nutritional screening in this patient group. This is not surprising, as all nutrition screening tools have their individual strengths and limitations and measure somewhat different aspects of nutritional status [62,63,64].

### 10.2. Diagnosis of Malnutrition

**Figure 3:**
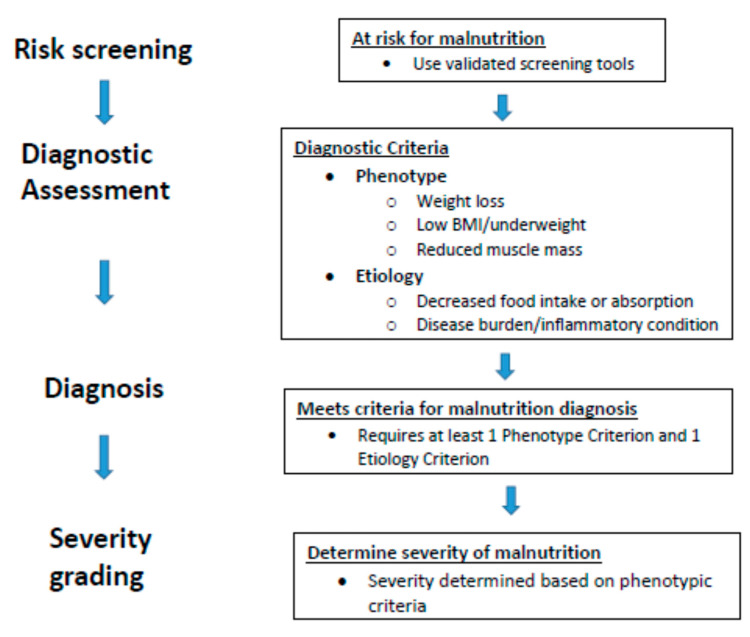
Global Leadership Initiative on Malnutrition (GLIM) diagnostic scheme for screening, assessment, diagnosis, and grading of malnutrition [40].

To respond to the need for a global consensus for defining and characterizing malnutrition in the clinical nutrition and medical communities, the Global Leadership Initiative on Malnutrition (GLIM) was established in 2016 [40] by the global clinical nutrition societiesAmerican Society of Parenteral and Enteral Nutrition (ASPEN) (www.nutritioncare.org) (accessed on 13 April 2021), the European Society for Clinical Nutrition and Metabolism ESPEN (www.espen.org) (accessed on 13 April 2021), Latin American Society of Parenteral and Enteral Nutrition (FELANPE) (www.felanpeweb.org) (accessed on 13 April 2021) and The Parenteral and Enteral Nutrition Society of Asia (PENSA) (www.pensa-online.org) (accessed on 13 April 2021). GLIM has agreed on a set of diagnostic criteria that is based on a process in two steps, starting with risk screening to identify at-risk status [40], as shown in Figure 3. Screening could be performed by different validated screening tools, such as the Nutrition Risk Screening 2000 (NRS-2000), Minimal Nutrition Assessement- Short Form (MNA-SF), Malnutrition Universal Screening Tool (MUST) and Subjective Global Assessment (SGA) [65,66,67,68]. The second step seeks to diagnose the condition and grade its severity. To make the diagnosis, there are three phenotypic criteria (weight loss (%), low body mass index (kg/m^2^), and reduced muscle mass) and two etiologic criteria (reduced food intake or assimilation and the presence of inflammation. The diagnosis requires that at least one phenotypic and one etiologic criterion is fulfilled, meaning that for different individuals, the possible combination of criteria may vary. The severity of the condition is categorized into Stage 1 (moderate malnutrition) and Stage 2 (severe malnutrition) depending on whether two phenotypic criteria are met. 

### 10.3. Nutritional Treatment of COVID-19 Patients

Evidence-based nutrition therapy specifically adapted to COVID-19 is limited. The European Society for Clinical Nutrition and Metabolism (ESPEN) has published expert statements and practical guidance for the nutritional management of COVID-19 patients [38]. ESPEN emphasized the importance of routine management including prevention, diagnosis, and treatment of malnutrition in these patients. Ten practical recommendations were proposed focusing those in the ICU setting or in the presence of older age and poly-morbidity, both of which are independently associated with malnutrition and negatively impact survival.

The European Federation of Dietetic Associations (EFAD) published a briefing paper that described the role of the dietetic profession in COVID-19 care [69]. The EFAD recognized that most attention has been directed at the hospitalized COVID-19 patient in the most acute phase. Those who have suffered from COVID-19 and have been hospitalized with an increased risk of malnutrition have likely experienced loss of muscle mass, and, regardless of the care setting, likely experienced malnutrition, changes in eating patterns, loss of sense of taste and smell, and a poor appetite in relation, all of which may directly impact recovery and rehabilitation. The EFAD therefore emphasized that even when people are well enough to leave hospital, their journey to return to health is not over; so, EFAD underlined the importance of continued nutrition care in primary care and rehabilitation.

The Academy of Nutrition and Dietetics (AND) in the U.S. published general guidance and practice considerations for registered dietitian nutritionists (RDNs) [70]. The AND noted that those with multiple comorbidities, who are older, and malnourished are at an increased risk of being admitted to the ICU and have increased rates of mortality from COVID-19. Therefore, nutrition care to identify and address malnutrition is critical in treating and preventing further adverse health outcomes. The importance of nutrition care throughout the patient’s journey from hospital to home care settings was emphasized. Furthermore, AND highlighted the importance of collaboration in multidisciplinary teams to manage malnutrition in these patients [71].

All three nutrition organizations agree on recognizing malnutrition as the main nutritional problem in COVID-19 patients and agree on the importance of a structured nutrition care plan for recovery. They complement each other, with ESPEN focusing on the ICU setting, while the EFAD and AND take a broader perspective underlining the importance of continued nutrition care after hospital discharge and through rehabilitation.

### 10.4. Vitamin D

In addition to the basic nutritional treatment of malnourished COVID-19 patients, specific micronutrients may also be of interest. A recent review discussed the potential role of several micronutrients and bioactive substances in the immune system [72]. Vitamin D is the single nutrient that, so far, has attracted the most interest in the COVID-19 literature, due to its role in the immune system [73,74]. Vitamin D is involved in the mechanisms of regulation of the immune system; it regulates the action of suppressor T lymphocytes, the synthesis of cytokines, and acts by modulating the processes of cellular apoptosis [75]. Respiratory monocytes, macrophages, and epithelial cells constitutively express the vitamin D receptor, which might therefore play a protective roll against respiratory infections through this receptor [74].

In a recent basic review including 47 papers, the hypothesis that vitamin D deficiency increases with COVID-19 rates and illness severity was studied [76]. The findings were not conclusive. The authors concluded that recommending 2000 IU daily for populations with limited access to vitamin D from the sun has no potential harm and might save many lives. This is in line with recommendations from the National Institute for Health and Care Excellence (NICE). NICE published an evidence review titled Vitamin D for COVID-19 in late June 2020 on the effectiveness and safety of supplementation for the treatment or prevention of, or the susceptibility to, COVID-19 based on vitamin D status [77]. Out of 187 papers, NICE analyzed 5, all with a high risk of bias. On September 20, a commentary on important new evidence that presented data from one new pilot randomized clinical trial (RCT) [78] was published. It was concluded that the RCT had several confounders, and so results should be interpreted with caution. Therefore the state of evidence for clinical management of COVID-19 patients did not change [79].

Some new observational studies [80,81,82] have been published after the NICE evidence review. Findings report a negative correlation between vitamin D status and COVID-19 severity, and in one study, in a negative correlation between vitamin D status and to mortality. However, these studies also suffered from different limitations and confounders.

Based on the present literature, evidence for vitamin D supplementation in COVID-19 patients still is limited and inconclusive.

## 11. Conclusions

The COVID-19 infection has hit older adults harder in terms of prevalence, severity, and outcomes. This is linked to a combination of age-related immunological and metabolic alterations. However, in the literature, documentation on nutritional status and management is limited. No studies with only people aged 65+ years were found, and documentation on the oldest population group (80+ years) is rare. The studies found showed high prevalence of malnutrition in the severely- and critically-ill patients. All seven considered the need for nutrition screening, management, and support, including as part of the follow-up management after hospital stay. Diagnosing malnutrition according to the GLIM criteria is recommended but was not used in any of found studies. Documentation of olfactory and gustatory dysfunction in relation to nutritional status is missing in older adults. Vitamin D is the nutrient that has attracted the most documentation; however, evidence for the supplementation of COVID-19 patients still is limited and inconclusive.
